# Anomalous Right Coronary Artery: A Multimodality Hunt for the Origin

**DOI:** 10.1155/2011/286598

**Published:** 2011-07-20

**Authors:** J. Gilmour, H. Kafka, G. Ropchan, A. M. Johri

**Affiliations:** ^1^Division of Cardiology, Queen's University, Kingston, ON, Canada K7L 3N6; ^2^Department of Radiology, Quinte Healthcare Corporation, Belleville, ON, Canada K8N 5A9; ^3^Division of Cardiac Surgery, Department of Surgery, University of Toronto, Toronto, ON, Canada M5G 1X8

## Abstract

Anomalous origin of the right coronary artery from the pulmonary artery (ARCAPA) is a rare congenital anomaly. Although there have been several cases of ARCAPA reported in the literature, we present a case which highlights the challenges of diagnosing this rare condition and the incremental value of using multiple imaging modalities. A healthy 48 year old female presented with angina and exertional shortness of breath. She had a normal cardiovascular examination, negative cardiac enzymes and an unremarkable chest X-ray. She did, however, have T-wave inversions in leads V1–V3. Transthoracic echocardiography (TTE), as the first imaging investigation, led to an initial provisional diagnosis of a coronary-cameral fistula. It showed unusual colour Doppler signals in the right ventricle and a prominent pattern of diastolic flow within the right ventricular myocardium, especially along the interventricular septum. A subsequent multimodality approach, correlating images from angiography, CT and MRI was instrumental in confirming the diagnosis of ARCAPA and planning for surgical correction. Cardiac CT and MRI are non-invasive, three-dimensional imaging modalities with high diagnostic accuracy for coronary artery anatomic anomalies. If echocardiography and conventional angiography have been inconclusive, cardiac CT and MRI are especially important diagnostic tools.

## 1. Introduction

Congenital anomalies of the coronary arteries occur in approximately 1% of the general population [1]. Anomalies can involve the origin, course, and/or termination of the vessel(s). Anomalous origin of the right coronary artery from the pulmonary artery (ARCAPA) is very rare and has an incidence of only 0.002%  [2]. ARCAPA has a broad range of clinical manifestations ranging from sudden death, chest pain, and shortness of breath to incidental discovery in asymptomatic individuals.

## 2. Case Report

A previously healthy 48-year-old female presented to the emergency department with sudden onset right scapular and right thoracic chest pain, as well as shortness of breath with exertion. She had no previous cardiovascular history and her only cardiac risk factor was a four pack-year history of smoking. 

On physical examination, her vital signs and cardiovascular examination were normal. There was no evidence of congestive cardiac failure or murmurs. She had tenderness to palpation inferior to her right scapula. Shoulder examination was otherwise normal. Serial cardiac enzymes were negative; however, her resting ECG showed T-wave inversions in leads V1–V3. Her chest X-ray did not show cardiomegaly or signs of pulmonary edema.

In the emergency department, it was suspected that her scapular pain was musculoskeletal in nature, as there was a clear history of repetitive strain and the pain responded to simple analgesics. On the other hand, her thoracic chest pain, exertional symptoms, and nonspecific T wave changes were still unexplained. 

Upon further questioning, she reported a similar acute presentation of right thoracic chest pain two years ago while visiting overseas. She had a brief hospital stay at the time; however, a definitive diagnosis was not made. Since that time, she had described frequent episodes of shortness of breath and thoracic chest discomfort precipitated by moderate exercise and relieved within five minutes of rest. Given the potential ischaemic nature of these symptoms, her primary care physician had previously referred her for formal cardiac investigation. An exercise-stress test had been inconclusive and a dobutamine stress echo (DSE) was negative for ischaemia. 

**Figure 1:**
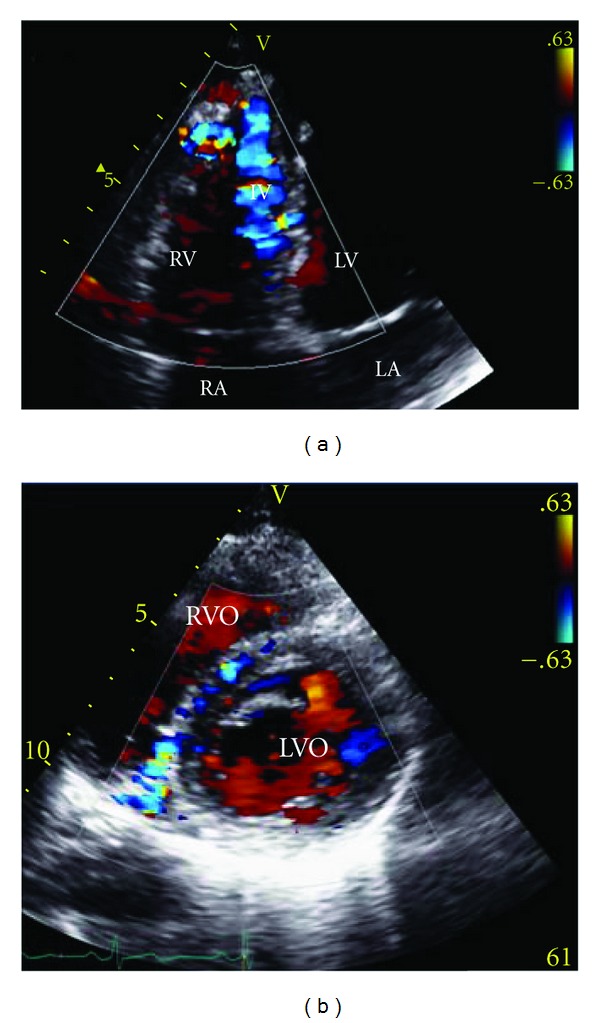
Transthoracic colour-flow Doppler echocardiography. (a) Four-chamber view. (b) Parasternal short axis view. Both images show multiple collateral vessels within the IV septum and abnormal signals in the coronary arteries, indicating dilatation and tortuosity. RA: right atrium; LA: left atrium; LV: left ventricle; RV: right ventricle; RVO: right ventricle outflow; LVO: left ventricle outflow; IV: interventricular.

While in hospital, a standard transthoracic echocardiogram (TTE) was performed; it showed normal LV size and systolic function, no valvular abnormalities, and no evidence of diastolic dysfunction. The TTE was remarkable for very unusual colour Doppler signals in the right ventricle (Figures 1(a) and 1(b)). There was a prominent pattern of diastolic flow within the right ventricular myocardium, especially along the interventricular septum. It was hypothesized that this flow was within dilated and tortuous coronary arteries. The differential diagnosis included a coronary-cameral fistula involving the right ventricle (RV) and ALCAPA (anomalous left coronary artery from the pulmonary artery). At the time, ALCAPA was thought to be an unlikely diagnosis as the direction of flow through the left coronary artery (LCA) was normal.

**Figure 2:**
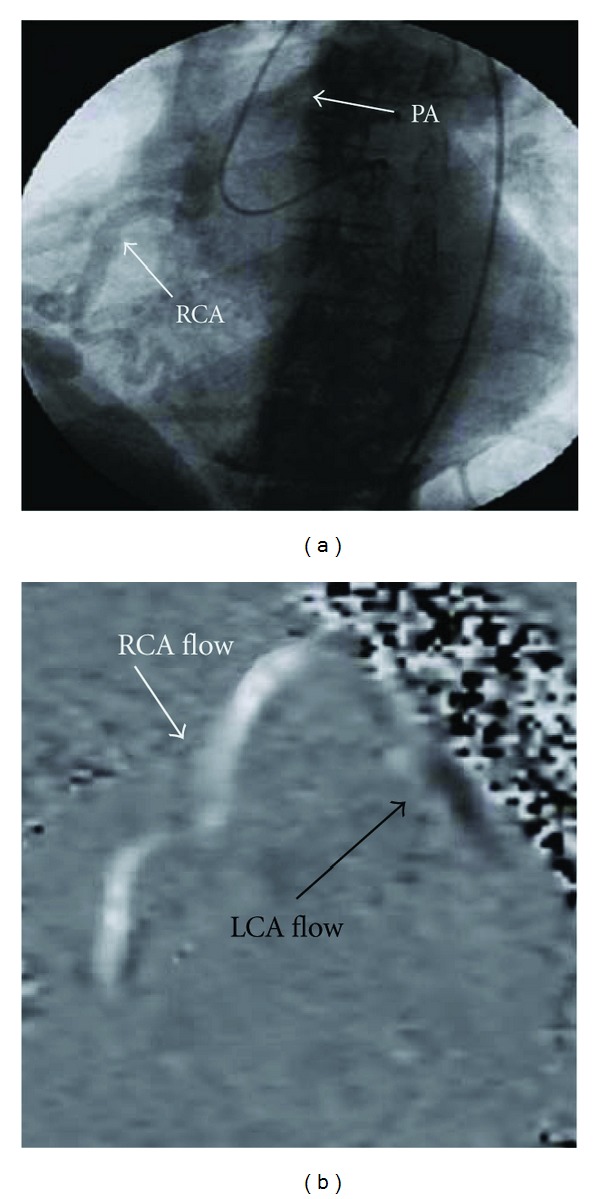
(a) Coronary angiogram. The catheter is in the aorta and the left main is engaged. Collateral vessels from the LCA deliver contrast media into the RCA, which subsequently drains into the main PA. (b) MRI velocity flow map. Coded black is flow down the LCA away from the aorta. Coded white is flow up the RCA towards the main PA. PA: pulmonary artery; RCA: right coronary artery; LCA: left coronary artery.

To clarify this woman's coronary anatomy, a standard coronary angiogram was performed. The pattern of blood flow was from the aortic sinus of Valsalva into the LCA, which then filled the right coronary artery (RCA) through collateral epicardial vessels; flow then passed into the pulmonary artery (PA) in a retrograde manner (Figure 2(a)). The possibilities were of a congenital RCA to PA fistula or a congenital anomaly with the right coronary artery originating from the proximal pulmonary artery. 

**Figure 3:**
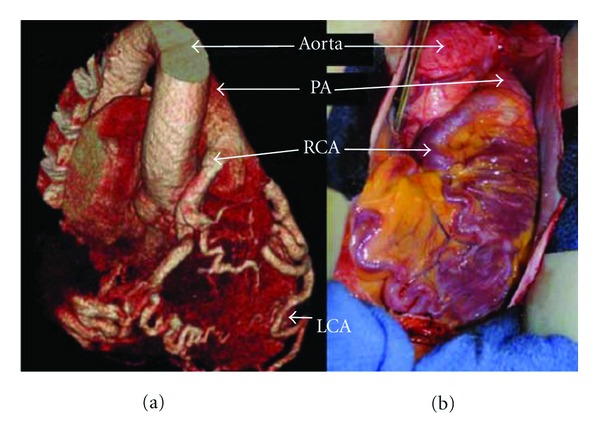
(a) Three-dimensional rendering of CT angiogram demonstrating ARCAPA. The right coronary artery originates from the anterior, lateral aspect of the pulmonary artery approximately 8 mm above the pulmonary valve. (b) intraoperative images of ARCAPA, prior to surgical correction. PA: pulmonary artery; RCA: right coronary artery; LCA: left coronary artery.

To enable more specific anatomical localization of the coronary circulation and heart structure, multislice computed tomography (MSCT) (Figure 3(a)) was performed. This investigation confirmed that the cardiac chambers were not dilated and there were no structural valve abnormalities. The left main coronary artery was seen to arise from its expected position in the left sinus of valsalva and to have a diameter of 9 mm at its origin; the left anterior descending and the proximal circumflex arteries were also substantially dilated. The dilated RCA (10 mm at its largest) did not originate from the right sinus of Valsalva; it was shown to originate from the antero-medial aspect of the main PA, approximately 8 mm above the pulmonary valve annulus. The RCA then coursed down anterior to the right sinus, traveled under the right atrial appendage into the AV groove, and then followed its normal course on the inferior wall of the heart. These findings were consistent with anomalous right coronary artery from pulmonary artery (ARCAPA). Cardiac magnetic resonance (CMR) (Figure 2(b)) was subsequently undertaken to enable flow study calculation; the flow from the RCA into PA occurred at an elevated rate of 1.3 L/min, which represented almost a quarter of her cardiac output. The finding of ARCAPA in this woman was not suspected after the initial imaging with TTE but was clarified by subsequent imaging with angiography, CT, and MRI.

Cardiac surgery was consulted. Based on her clinical symptoms and review of the available literature, the decision to proceed to surgical repair was undertaken. It was thought that it would be feasible to transect the RCA, ligate the stump at its origin on the PA and anastomose the proximal RCA to the anterior aorta. In the operating room, the exposed course of the anomalous right coronary artery was exactly as predicted by imaging (Figure 3(b)). The anatomic knowledge provided by multi-modality imaging a priori, allowed for the implementation of a surgical plan to restore right coronary artery circulation to its usual approximate origin. The patient had an uneventful postoperative course and convalescence; she was discharged with cardiac rehabilitation and yearly follow-up TTE.

Follow-up at one and two years revealed that this woman no longer experienced shortness of breath on exertion or chest discomfort. TTE showed no evidence of abnormality in cardiac function or structure. 

## 3. Discussion

ARCAPA was first described in 1885 by Brooks, who saw the anomaly in two separate cases at the time of autopsy [3]. Since this time, just over one-hundred cases have been documented, each varying in clinical presentation, demographic data, and imaging method used for diagnosis. Our case is the first to correlate images obtained from echocardiography, angiography, cardiac CT, CMR, and in the operating room. This multimodality approach was essential to developing an accurate diagnosis and planning treatment. 

Although ARCAPA is rare, it can be a serious condition. The most common clinical presentation is angina [4]; however, a number of other presentations have been observed, including sudden death, congestive cardiac failure, arrhythmias, and myocardial infarction. ARCAPA can be an isolated anomaly or can be associated with other congenital heart defects, such as aorticopulmonary window or Tetralogy of Fallot. Generally individuals with isolated ARCAPA commonly present in adult life. This case depicts a typical presentation of ARCAPA, as an isolated anomaly presenting in an adult with both angina and dyspnoea. 

Prior to 1965, all diagnoses of ARCAPA were made during surgery or at autopsy [4]. Cardiac imaging has advanced since this time and a variety of imaging modalities have been employed in arriving at a diagnosis; TTE and conventional coronary angiography have been the most popular methods to date. The diagnosis of ARCAPA, in this case, was made with the use of multiple cardiac imaging modalities, each having its own advantages, risks, and limitations.

The initial imaging investigation was TTE with Doppler studies; this demonstrated abnormal flow within the coronary arteries but was unable to delineate the actual coronary anatomy present in the individual. In the literature, the most consistently recorded echocardiography findings for ARCAPA are the presence of intercoronary collateral blood flow within the ventricular septum and a dilated LCA/RCA [5]. When reconsidered in hindsight, it is clear that the flow seen within the tortuous coronary vessels on the surface of the RV incorrectly gave the appearance of a coronary-cameral fistula. Other cases of ARCAPA have also been initially misdiagnosed with a two-dimensional echocardiography, including a recent case described by Kühn et al., in which a VSD was incorrectly suggested [6]. In cases of coronary artery abnormalities, the course and origin of each vessel can be complex and therefore, not clearly represented with two-dimensional images. Both MSCT and CMR are better noninvasive imaging investigations to allow three-dimensional representation of cardiac anatomy. CMR was performed later as a supportive investigation for the diagnosis of ARCAPA. CMR, like MSCT, allows for a three-dimensional representation of the coronary anatomy and in addition can determine blood flow direction and velocity. CMR is also advantageous in that it does not require ionizing radiation or intravenous contrast medium.

Both CMR and MSCT are becoming increasingly popular imaging modalities for detecting coronary artery abnormalities. Even in cases where a definitive diagnosis of ARCAPA can be made with echocardiography or conventional angiography, MSCT and/or CMR is recommended to allow more definite spatial resolution in anticipation for surgery.

## 4. Conclusion

MSCT and CMR are noninvasive, three-dimensional imaging modalities with high diagnostic accuracy for coronary artery anatomic anomalies. If echocardiography and conventional angiography have been inconclusive, MSCT and CMR are especially important diagnostic tools.
